# Fluoroscopy-guided removal of a migrated non-palpable implanon adherent to the ulnar nerve: a case report

**DOI:** 10.3389/fmed.2026.1801202

**Published:** 2026-05-29

**Authors:** Changsung Han, Chungwon Lee

**Affiliations:** Department of Thoracic and Cardiovascular Surgery, Pusan National University School of Medicine, Biomedical Research Institute, Pusan National University Hospital, Busan, Republic of Korea

**Keywords:** contraceptive implant, etonogestrel, fluoroscopy, foreign body, ulnar nerve

## Abstract

Although subdermal contraceptive implants are highly effective, they can create various complications if they migrate or become non-palpable, especially when adjacent to neurovascular structures. Ultrasound guidance and hydrodissection are commonly used to remove migrated implants; however, these techniques are not practical in certain cases. We report the case of a 33-year-old left-handed woman who presented with persistent abnormal bleeding 3 years post-etonogestrel implant placement. The implant was non-palpable upon presentation, and previous removal attempts had failed. Imaging revealed that the implant was located deep in the upper arm, adjacent to the ulnar nerve. Ultrasound guidance and hydrodissection were unsuccessful; therefore, fluoroscopy guidance was used to safely extract the implant, which had adhered to the ulnar nerve. The patient was discharged on the same day. By the 2-week follow-up visit, the incision had healed well, and the patient reported that the transient tingling sensation—which had occurred intraoperatively due to minimal traction during the final dissection—had completely resolved. Fluoroscopy-guided implant removal is effective for the removal of deeply placed or migrated implants, particularly when conventional methods fail; however, accurate localization and referral to specialists are essential for their safe removal.

## 1 Introduction

Subdermal etonogestrel-releasing implantable contraceptives are widely used, owing to their high efficacy, reversibility, and convenient long-acting nature. The implant is a single 4–5 cm × 0.5-cm flexible rod typically inserted in the medial subdermal aspect of the upper arm, approximately 8 cm above the medial epicondyle. It contains 68 mg of etonogestrel and remains effective for approximately 3 years in the majority of cases, although it can last for up to 4 or 5 years in some cases.

As the use of these implants has increased, complications related to deep insertion, fracture, and migration have become more frequent (1). Approximately 3–5% of implants are inserted too deep, become non-palpable, or migrate from their original subdermal placement. Migration into or adjacent to neurovascular structures, which may lead to neuropathy, vascular injury, intravascular migration, and/or, although exceptionally rare, pulmonary embolization of the implant, is of particular concern (2). While standard protocols for managing difficult implant removals have been established to improve safety and efficacy (3), non-palpable and migrated implants still present significant technical challenges necessitating image-guided localization for safe retrieval (4).

In this study, we report a rare case of a non-palpable etonogestrel implant that migrated and subsequently adhered to the ulnar nerve in the upper arm. After unsuccessful conventional ultrasound-based removal attempts, the implant was successfully removed under fluoroscopic guidance. This case highlights the important technical factors to be considered when managing contraceptive implant migration within high-risk neurovascular regions.

## 2 Case description

**Figure 1:**
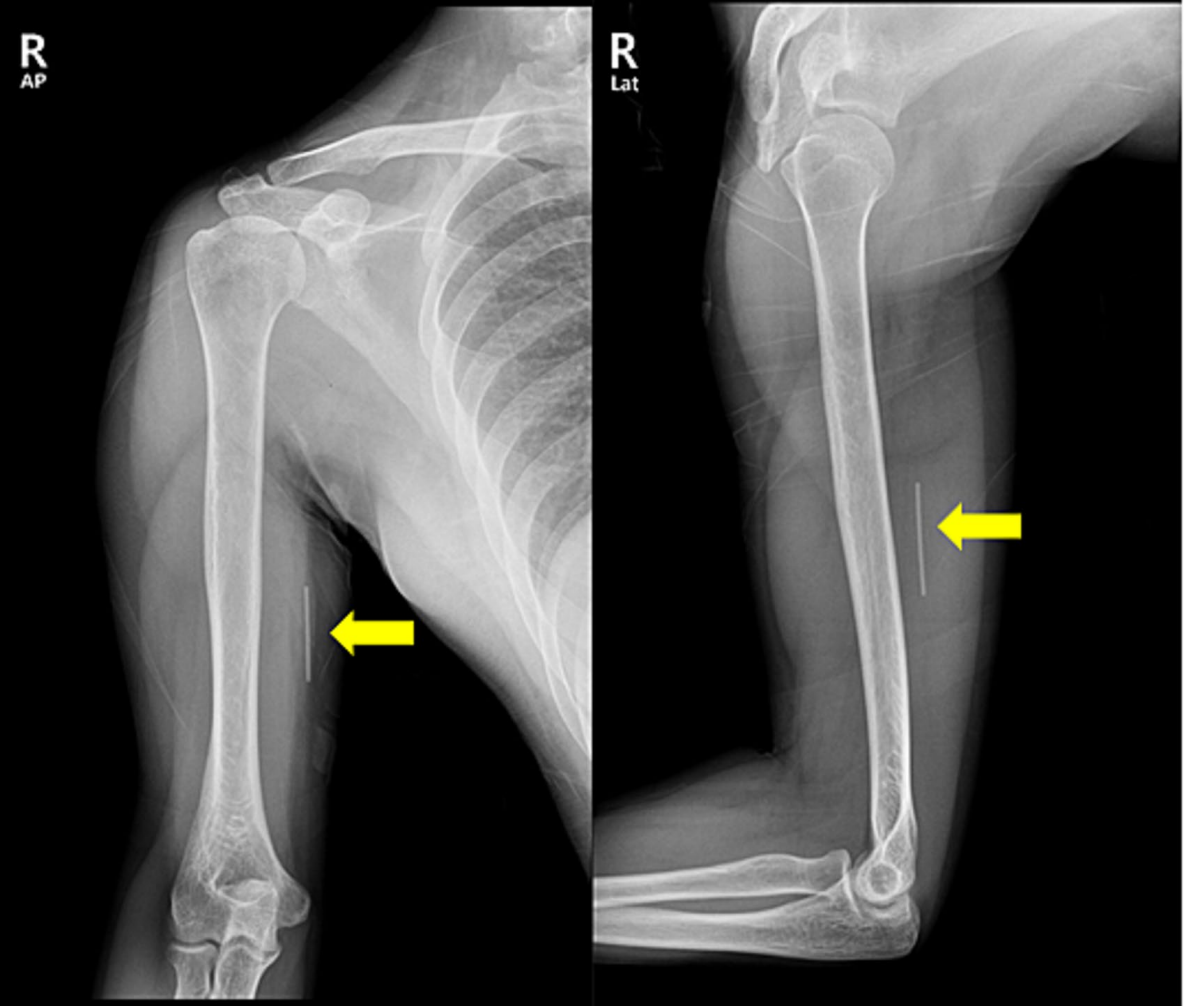
X-ray image of the etonogestrel implant (yellow arrow) positioned in the right upper arm of a 33-year-old woman (Busan, South Korea, 2025).

We report the case of a 33-year-old left-handed woman who presented with persistent abnormal uterine bleeding and future pregnancy planning 3 years after etonogestrel implant placement. Notably, the patient reported no pre-existing neurological complications, abnormal sensations, or nerve-related symptoms prior to the surgical intervention. The implant was non-palpable upon presentation. According to the patient’s medical history, two unsuccessful removal attempts had been made at a local clinic prior to her referral to our institution. This initial procedure was a blind attempt performed in a standard outpatient setting, where the clinician relied solely on the previous insertion site and simple palpation. However, the attempt failed because the implant was positioned deeply and had migrated from its original site. Upon referral to our institution, the obstetrics/gynecology department and orthopedic services were both unsuccessful at localizing the device. Given the suspected proximity of the implant to critical neurovascular structures, the patient was referred for consultation with a cardiothoracic surgeon (see Figure 1).

**Figure 2:**
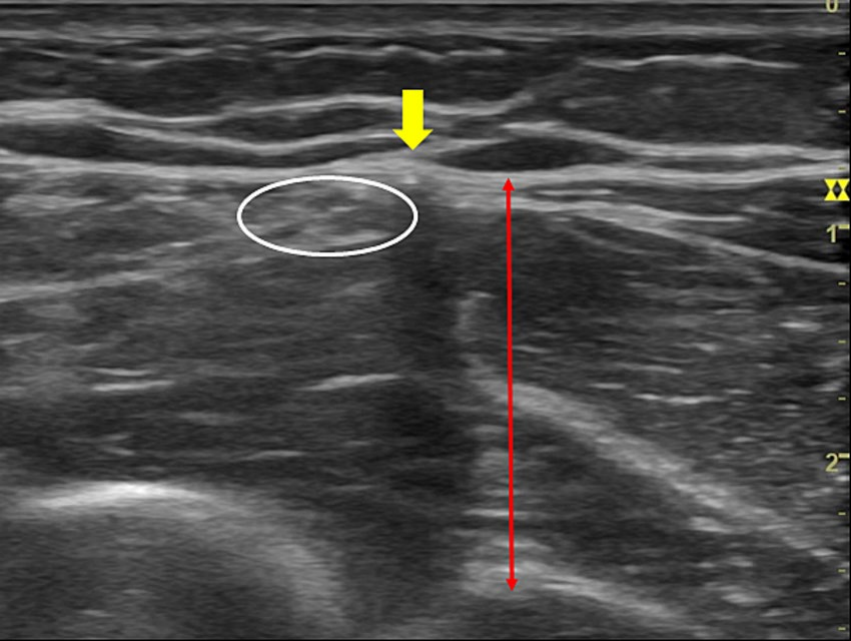
High-resolution ultrasound image of the non-palpable etonogestrel implant (yellow arrow) abutting the ulnar nerve sheath (white circle) in a 33-year-old woman (Busan, South Korea, 2025); bilateral red arrows indicate posterior acoustic shadowing beneath the etonogestrel implant. UN, ulnar nerve; US, ultrasound.

A physical examination of the patient’s arm revealed a healed scar from the previous removal attempts. Palpation again failed to identify the implant; however, high-resolution ultrasound images revealed that the implant was located deep within the subdermal layer of the medial upper arm, abutting the ulnar nerve sheath (Figure 2). No arterial or venous involvement was noted, although the interface between the implant and the nerve appeared indistinct. Given its non-palpable nature, prior failed attempts, and proximity to critical neuroanatomy, surgical removal of the implant was planned.

**Figure 3:**
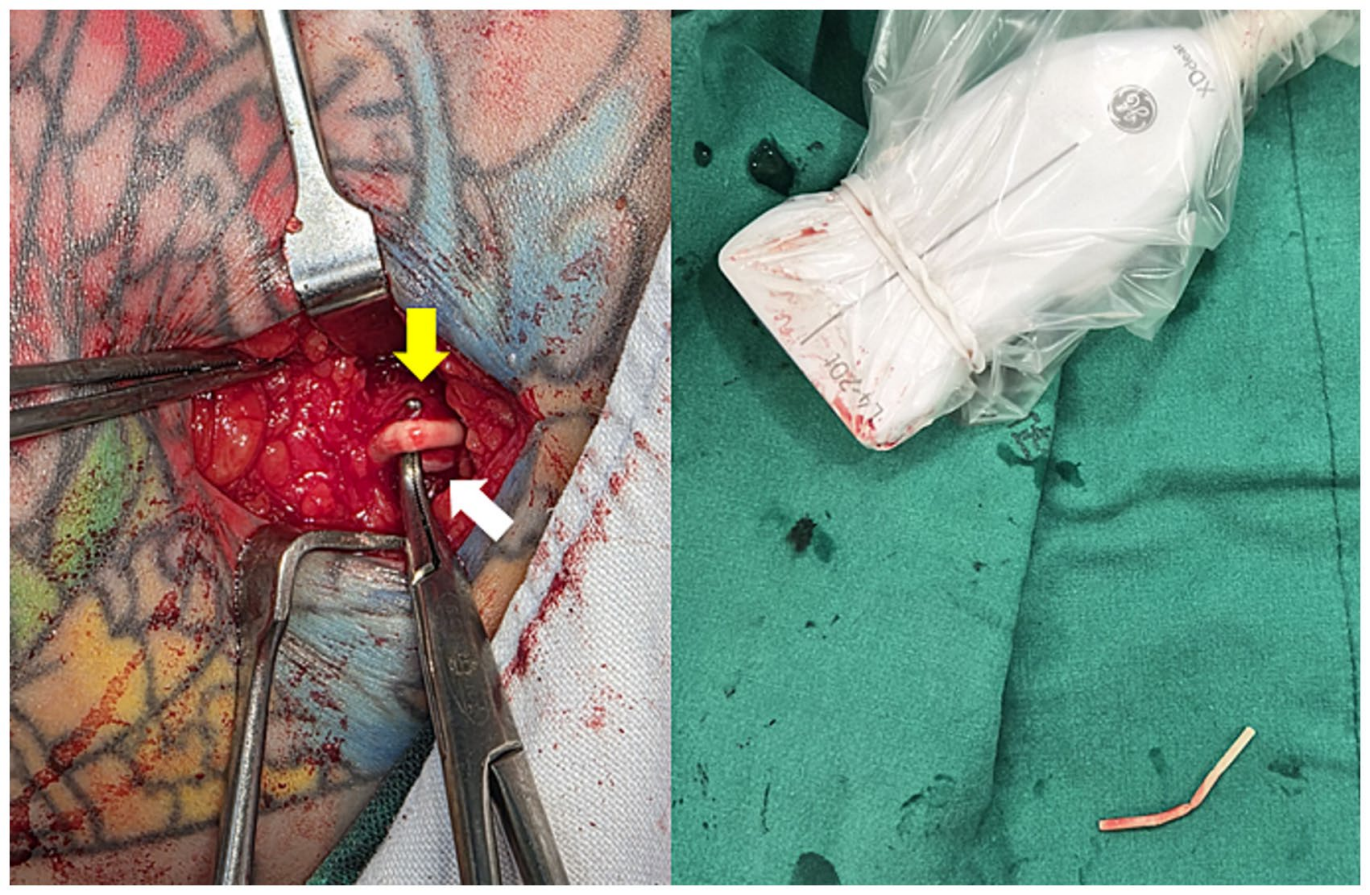
Intraoperative photograph of the surgical site (Busan, South Korea, 2025). The etonogestrel implant (yellow arrow) is presented, abutting the ulnar nerve (white arrow) and after successful retrieval as an intact rod from a 33-year-old woman (intact rod).

The surgical procedure was performed in the operating room. To optimize visualization and ensure surgical precision, the patient was positioned supine with the operative arm abducted on a dedicated arm table. The contralateral arm was maintained in a neutral adducted posture. Local anesthesia was achieved using 20 mL of 1% lidocaine diluted with normal saline. Notably, epinephrine was excluded to avoid potential vasoconstriction-induced ischemia and to maintain optimal perfusion around the ulnar nerve. Drawing from our team’s extensive experience in vascular access surgery (e.g., arteriovenous fistula creation), a local field block was performed into the subdermal and fat layers. This approach was prioritized to facilitate continuous, real-time neurological monitoring through the patient’s verbal feedback, ensuring the safe separation of the implant from the ulnar nerve. The previous incision was reopened, and the suspected location was dissected (Figure 3). Although the implant was completely non-palpable, its location was identified via high-resolution ultrasound. Hydrodissection using 5% dextrose solution (D5W) was planned to create a safe dissection plane and protect the adjacent ulnar nerve. However, during the initial subcutaneous lidocaine injection for anesthesia, significant ultrasound artifacts occurred, obscuring the boundary between the implant and the nerve sheath. Furthermore, even minimal mechanical manipulation during the injection caused the implant to migrate further proximally, rendering real-time sonographic guidance unreliable. Consequently, the hydrodissection attempt was abandoned to prevent iatrogenic nerve injury, and the procedure was transitioned to fluoroscopy-guided removal. Fluoroscopy was then utilized to precisely locate the implant. During the procedure, approximately five intermittent fluoroscopic exposures were required. To minimize radiation exposure for both the patient and the surgical team, we used intermittent fluoroscopy rather than continuous cine-fluoroscopy. Standard radiation protection protocols, including the use of lead aprons and thyroid shields, were strictly followed.

**Figure 4:**
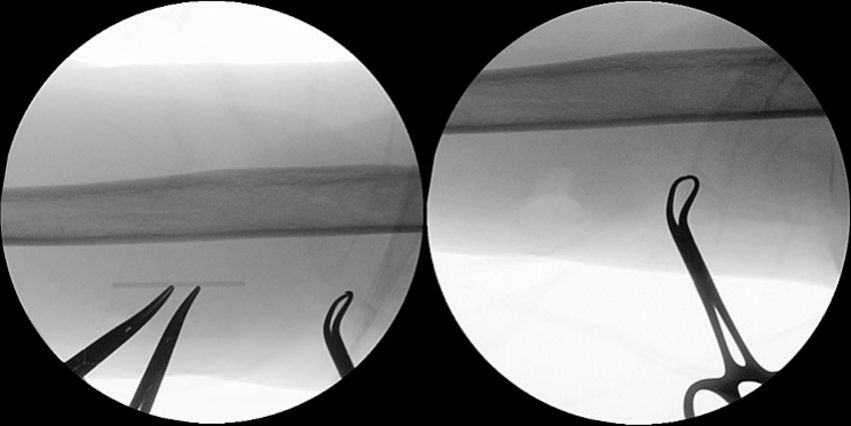
Successful retrieval of an intact 4 cm etonogestrel implant under fluoroscopic guidance after failed ultrasound-guided attempts in a 33-year-old woman (Busan, South Korea, 2025).

The prior incision was slightly extended, and blunt and sharp dissections exposed the implant deep in the fascia, adhering to the inferolateral aspect of the ulnar nerve. Traction produced radiating paresthesia along the ulnar distribution; therefore, gauze-assisted blunt dissection and limited sharp division of the adhesions allowed for gradual implant mobilization. The intact 4-cm rod was successfully removed under fluoroscopic guidance (Figure 4).

The incision was closed by Vicryl 3-0, and the patient was monitored postoperatively before being discharged the same day. At the 2-week follow-up, the patient’s incision had healed well. A transient tingling sensation occurred intraoperatively during the final stage of dissection, specifically when the implant—which was tightly adherent to the ulnar nerve sheath—was being separated. This sensation was likely attributable to the minimal traction required to release the adherent rod from the nerve. However, at the 2-week follow-up visit, the incision had healed well, and no abnormal sensations or neurological deficits were identified, confirming the resolution of the transient symptoms and the absence of permanent iatrogenic nerve injury (see Table 1).

**Table 1:** Timeline of the episode of care.

Time point	Key event
3 years before surgery	Subdermal etonogestrel implant placement at a local clinic
3 years after placement	Presentation with persistent abnormal uterine bleeding; request for removal
Prior to referral	Two unsuccessful blind removal attempts at a local clinic; localization failed
Upon referral to institution	Multidisciplinary consultation (OBGY, Orthopedics); localization confirmed via high-resolution ultrasound
Day 0 (surgery)	Attempted ultrasound-guided hydrodissection; transition to fluoroscopy-guided removal due to artifacts and intraoperative migration; successful retrieval of the intact 4-cm rod
Day 0 (post-op)	Same-day discharge after postoperative monitoring
Week 2 (follow-up)	Clinical evaluation: incision well-healed; complete resolution of intraoperative transient tingling; no permanent neurological deficits
Month 6 (follow-up)	Continued absence of neurological complications or long-term deficits; sustained clinical recovery

## 3 Discussion

The migration of subdermal contraceptive implants into neurovascular structures, although uncommon, is clinically significant due to the potential risk of neuropathy and the inherent technical challenges associated with their removal. Previous studies by Thibaut et al. (5) and Seo et al. (6) have established ultrasound guidance and hydrodissection as the gold standard for managing non-palpable implants. These techniques are designed to improve localization and create a safe dissection plane, thereby minimizing the risk of iatrogenic injury. However, as demonstrated in our case, these methods are highly dependent on clear, stable sonographic visualization and the ability to accurately separate tissue planes. Our findings highlight a specific ‘technical gap’ encountered when an implant is tightly adherent to neurovascular structures or exhibits significant intraoperative mobility, rendering standard ultrasound-based approaches unreliable.

In our patient, the firm adherence to the ulnar nerve sheath, combined with ultrasound artifacts and proximal migration induced by the initial anesthetic injection, resulted in significant positional shifts. These factors hindered the clinician’s ability to distinguish the implant from the surrounding nerve tissue in real time. In this context, fluoroscopy-guided removal serves as a vital ‘next-step’ rescue strategy when the conventional ‘ultrasound-first’ approach reaches its technical limits. Unlike ultrasound, which can be obscured by air bubbles or fluid-induced artifacts, fluoroscopy provides superior, continuous positional visualization and improved control. It allows for the immediate detection of unexpected implant movements, which is particularly crucial when the foreign body is positioned in high-risk anatomical zones near major nerves or vessels. In this regard, awake neurological monitoring via continuous verbal feedback served as a critical safety mechanism, compensating for the lack of real-time nerve visualization under fluoroscopy. According to the protocol for difficult implant removals, ultrasound-guided localization remains the primary approach. However, as Voedisch and Hugin (3) emphasized, a transition to alternative imaging or a multidisciplinary approach is indicated when conventional visualization is compromised. Although cross-sectional imaging such as magnetic resonance imaging (MRI) or computed tomography (CT) can provide detailed anatomical maps, they were not performed in this case, as high-resolution ultrasound already provided sufficient detail for localization relative to the ulnar nerve. Furthermore, this decision was part of a shared decision-making process; the patient preferred to avoid additional high-cost imaging that was unlikely to alter the immediate surgical plan given the definitive sonographic findings. This approach aligns with a cost-effective, patient-centered rescue strategy without compromising procedural safety. In our case, the occurrence of ultrasound artifacts and intraoperative migration served as the specific clinical indicators for transitioning to fluoroscopy.

Even minor mechanical pressure from hydrodissection or manual manipulation can induce displacement beyond expected limits, potentially leading to further complications if not monitored continuously. Therefore, we propose that a multidisciplinary approach is essential for complex removal cases. Early recognition of the technical limitations of ultrasound and a timely transition to fluoroscopic guidance—coordinated by clinicians with extensive expertise in upper extremity neurovasculature—are critical steps for reducing procedural risks. By integrating fluoroscopy as a reliable alternative, surgeons can achieve successful retrieval while ensuring maximal preservation of neurological function and improving overall patient outcomes.

### 3.1 Limitations

A primary limitation of this case report is the short follow-up duration (2 weeks). While a 3–6 month observation is ideal to definitively rule out delayed neuropathy, the patient reported complete symptom resolution and did not return despite explicit instructions to report any new neurological deficits. Although spontaneous non-attendance often suggests successful recovery and patient satisfaction in clinical practice, we acknowledge that long-term objective assessments would further validate the permanent safety of this fluoroscopy-guided approach.

## Data Availability

The raw data supporting the conclusions of this article will be made available by the authors, without undue reservation.
